# Linear and Nonlinear Effects in Connectedness Structure: Comparison between European Stock Markets

**DOI:** 10.3390/e24020303

**Published:** 2022-02-21

**Authors:** Renata Karkowska, Szczepan Urjasz

**Affiliations:** Faculty of Management, University of Warsaw, Szturmowa Street 1/3, 02-678 Warsaw, Poland

**Keywords:** stock market, market connectedness, mutual information, transfer entropy, COVID-19, crisis

## Abstract

The purpose of this research is to compare the risk transfer structure in Central and Eastern European and Western European stock markets during the 2007–2009 financial crisis and the COVID-19 pandemic. Similar to the global financial crisis (GFC), the spread of coronavirus (COVID-19) created a significant level of risk, causing investors to suffer losses in a very short period of time. We use a variety of methods, including nonstandard like mutual information and transfer entropy. The results that we obtained indicate that there are significant nonlinear correlations in the capital markets that can be practically applied for investment portfolio optimization. From an investor perspective, our findings suggest that in the wake of global crisis and pandemic outbreak, the benefits of diversification will be limited by the transfer of funds between developed and developing country markets. Our study provides an insight into the risk transfer theory in developed and emerging markets as well as a cutting-edge methodology designed for analyzing the connectedness of markets. We contribute to the studies which have examined the different stock markets’ response to different turbulences. The study confirms that specific market effects can still play a significant role because of the interconnection of different sectors of the global economy.

## 1. Introduction

Correlation estimates are crucial not only for asset allocation decisions but also for risk management and hedge. Following the global financial crisis (GFC), we have another critical period in the financial market—global outbreak of the coronavirus (COVID-19) [1]. The pandemic is influencing a number of channels, including commercial activities, consumption, labor markets, and international supply chains. Among these channels, one of the most important components is the stock markets [2,3].

As a result, investors are more active and efficient in transferring their investments from one market to another in the event of a financial crisis, particularly at the first signs of economic or political instability. However, at a time when financial crises and pandemic turbulences are systemic in nature, the process of international diversification of assets may not fulfill its basic role—risk reduction. Additionally, empirical studies confirm that correlations between markets change over time, which makes the benefits of the theory of diversification of investment portfolio selection questionable [4]. The main goal of this paper is to verify the risk transfer between US stock market indices and six European stock market indices under the 2007–2009 global financial crisis and COVID-19 outbreak.

In our study, we compare the Central and Eastern European (CEE) and Western European markets, even though these countries are forming a common area of the European Union together. The motivation to perform this division is to compare markets from countries with different levels of economic development, including the financial market. Keeping this in mind, the risk transfer structure may be different for these two regions. Our previous research confirms this relationship [5]. Our interest in that group of countries stems from several insights. Firstly, CEE countries have made major structural changes and reforms to integrate into European structures. Therefore, verification of how the financial markets of transition countries interact with other markets is an interest for both policy makers and investors. Secondly, CEE countries offer high returns on capital market investments with relatively low risk. Additionally, as the financial systems of CEE countries are strongly bank-based, an analysis of stock market development may still provide useful information.

The main contributions of this paper could be capitulated as follows. Firstly, we contribute to the studies which have examined the different stock markets’ response to different turbulences (financial crisis and pandemic outbreak). Thus, we answer the question whether they can be equally responsible for the intensification of the impact of the US stock market on the stock exchanges of Central and Eastern Europe. Secondly, we employ a variety of methods to separately analyze the linear and nonlinear effect of connectedness structures for international equity markets. The area of transfer entropy has not been explored in depth. Therefore, using linear and nonlinear methodology, we can compare the complexity of the behavior of stock markets. Interesting results were obtained by Olbryś and Majewska [6], who examined the benefits of diversifying their international portfolio to the largest European stock markets (i.e., the UK, France, and Germany) during the period 2003–2013. To the best of our knowledge, no current study has analyzed connectedness structures by verifying the linear and nonlinear effect in CEE stock markets compared to Western European markets during the COVID-19 pandemic.

Thirdly, we can observe that the correlations between US and other European markets are unstable. Additionally, we confirm that Western European markets displayed higher results of the correlations with the US stock market in comparison to CEE [7].

Fourthly, the study emphasizes that while globalization has contributed to a more integrated financial system, specific market effects can still play a significant role because of the interconnection in different countries of the global economy. From an investor perspective, our findings suggest that in the wake of the global crisis and pandemic outbreak, the benefits of diversification will be limited by the transfer of funds between developed and developing country markets.

The analysis by Gao and Mei [8] examined the structure of the correlation between the US and Asian stock indices during the global financial crisis of 2007–2009 with the use of a sliding window. As part of our article, we carried out verification of the method used by Gao and Mei [8] in relation to European indices, extending the research sample to the period of the COVID-19 pandemic. The sliding window is a technique used by [8,9,10] to obtain dynamically changing results in observation windows. Using various parameters of sliding windows allowed for receiving distinctive outputs that presented slightly different trends in the time series. Using the methods of linear correlations, mutual information, and transfer entropy, which take into account the sliding window, it was possible to build a network of risk transfer structure relationships for the daily rates of return of selected Western European markets and Central and Eastern European equity markets. We show that these networks detect significant differences in the behavior of individual stock indices, especially in turbulent market periods, thus highlighting the strongly changing relationships between stock markets in different countries.

The rest of the paper is organized as follows. Section 2 presents the literature review, while Section 3 provides the description of the data. Section 4 presents methodology. Section 5 analyzes the results of the linear and nonlinear effect in connectedness structures. Finally, Section 6 concludes with some discussion regarding the implications of the findings and possible extensions to future work.

## 2. Literature Review

Although there is no consensus in studies on the reasons for increasing inter-market correlations in times of market turbulences, most researchers accept that correlations change fundamentally during market crises. The empirical results of Boubaker and Raza [4] provide strong evidence of cross-market movement between US and CEE stock markets and show that joint movement exhibits large time differences and asymmetry in the tails of return distributions. The analysis demonstrated that changes in volatility in the US and the euro area are relevant factors causing risk shocks in European markets.

Studies on the impact of COVID-19 on the financial market spread rapidly; however, they still do not cover all economic aspects of the pandemic. The overall economic impacts are not yet straight, and there is no consensus in the research. For example, Ashraf [11], Zhang et al. [12], Akhtaruzzaman et al. [13], and Zaremba et al. [14] confirm that the last pandemic has led to a growth in global financial market risk. On the other hand, Sharif et al. [15] indicate that the COVID-19 pandemic affects the US economic risk much less than the geopolitical risk. Given a slower economic growth and relatively not liquid capital markets, it is possible that emerging markets have limited resources to cope with the pandemic. According to Topcu and Gulal [16], the negative impact of COVID-19 on emerging stock markets has gradually fallen and began to taper off by mid-April 2020. The recent result of the TGARCH model estimated in Visegrad group countries’ markets reveals that there is a negative link between the stock market indices and COVID-19 spread [17].

Even though the correlation coefficient and regression models are measures of linear relation between the markets, there are also nonlinear effects that may not be captured with the linear methods. The vast majority of research in transfer entropy estimation concerns developed markets. For example, Qiu and Yang [18] verify the estimation of transfer entropy for short time sequences, using 38 important stock market indices from four continents to create further financial networks, omitting nevertheless Central and Eastern European markets. Similarly, Kuang [19] aims to construct the information flow networks on multi-time-scales among 31 international stock markets between 2007 and 2018, finding that developed markets are more dominant but vulnerable to short-term risk contagion. An interesting study was conducted by Karaca, Zhang, and Muhammad [20] to optimize the stock indices’ forecasting model in the stock indices dataset; however, in their study, they used only the French and German indices. Nevertheless, developing stock market connectedness based on nonlinear methods such as mutual information and transfer entropy is still at a very early stage [21,22,23,24,25,26,27].

Mutual information and entropy transfer are frequently used methods to study the effect of long-memory volatility. Long-memory volatility can be seen as evidence of market participants’ inability to use the information available on the market and can, therefore, be linked to the issue of (not) market efficiency. For example, Dima and Dima [28] analyze the case of the Bucharest stock exchange, where they suspect endogenous and exogenous causes of nonlinear volatility effects. They suggest that mutual information can be an alternative method of checking persistence, which can be understood as evidence of long memory in the financial market. Caginalp and Desantis [29] emphasize that the role of long-term volatility is not the explicit opposite of a risk/return relationship but rather that there is an ambiguous and complex relationship between volatility and return. Khoojine and Han [30] used the mutual information method to build a structure describing the return and trading volume network of the Chinese stock. You, Fiedor, and Hołda [24] use mutual information to analyze the correlation structure of the stock market in Shanghai and find that the Chinese stock market is not structurally riskier than US and Western Europe markets. Barbi and Prataviera [21] study nonlinear dependencies on the Brazilian equity network and underline the particular benefit of mutual information network analysis to identify the characteristics of financial markets due to nonlinear relationships. Ferreira, Dionísio, Almeida, Quintino, and Aslam [31] review the influential dynamics of CEE stock indices as well as US, German, UK, and Chinese indices and find strongly influential correlations between some CEE indices and the impactful character of the US index. They argue that the COVID-19 pandemic could intensify the influence of Chinese and US indices.

Thus, we believe that there is a need for development of a study that provides an insight into the cutting-edge methodology for analyzing the connectedness of stock markets, together with a structural and time analysis of the stock exchange in CEE and Western Europe comparing the 2007–2009 financial crisis and the COVID-19 pandemic outbreak.

## 3. Data Characteristics

The data used in this study were taken from the Stooq website and consist of daily logarithmic returns of one US stock market index: SPX (S&P500 Index–New York) and six European market indices, of which three are from developed countries: UKX (FTSE 100 Index–London), CAC (CAC40 Index–Paris), DAX (DAX Index–Frankfurt), and three are from developing countries: WIG20 (WIG20 Index–Warsaw), PX (PX Index–Praha), BUX (BUX Index–Budapest). The allocation was made in accordance with the classification used by MSCI Inc. [32].

There are 4773 observations for each time series in the period between January 2000 and August 2020. Table 1 presents preliminary statistics of the daily logarithmic returns for all indices. The measure of skewness demonstrates that all-time series are skewed. On the basis of excess kurtosis, we can see that almost all series are highly leptokurtic with respect to the normal distribution. The Doornik–Hansen tests show a rejection (at the 5% level) of the null hypothesis of normality for each of the return series.

## 4. Methods

### 4.1. Cross-Market Correlations

As a first step, we use the Pearson correlation coefficient to measure the linear relationship. Next, we proposed an adjusted correlation coefficient following studies by Forbes and Rigobon [33], Olbryś and Majewska [6], and Rigobon [34]:

(1) $$\hat{\rho_{VA}}=\frac{\hat{\rho_{C}}}{\sqrt{1+\delta \left[1-{\left(\hat{\rho_{C}}\right)}^{2}\right]}}$$

where:

$\hat{\rho_{VA}}$—the adjusted correlation coefficient;

$\hat{\rho_{C}}$—the conditional (unadjusted) correlation coefficient;

δ—the change in turbulent period (crisis) volatility compared to the tranquil period (pre-crisis):

(2) $$\delta =\frac{{\hat{\sigma }}_{C}^{2}}{{\hat{\sigma }}_{PC}^{2}}-1$$

where ${\hat{\sigma }}_{C}^{2}$, ${\hat{\sigma }}_{PC}^{2}$ are the variances in the turbulent and tranquil periods.

Following that, the formula to transform Pearson correlations to a Fisher *Z* transformation is [35]:

(3) $$\rho_{VA}*=\frac{1}{2}\left[\ln\left(\hat{\rho_{C}}+1\right)-\ln\left(\hat{\rho_{C}}-1\right)\right]$$

To obtain approximately standard normal distributed *z*-statistic values, the difference is formed as follows:

(4) $$Z=\frac{\left(\rho_{C}-\rho_{PC}\right)}{\sqrt{\frac{1}{n_{C}-3}+\frac{1}{n_{PC}-3}}}$$

where $\rho_{C},\;\rho_{PC}$ are the cross-correlation coefficient in the turbulent and tranquil periods and $n_{C}$ and $n_{PC}$ are the sample sizes of the turbulent periods and tranquil period.

To verify the existence of significant change in cross-market correlations, we can test the hypotheses as follows:

(5) $$H_{0}:\rho_{VA}=\rho_{PC}\;H_{1}:\rho_{VA}\neq \rho_{PC}$$

where $H_{0}$ states that there are no significant changes in adjusted correlation.

### 4.2. Larntz–Perlman Procedure

We used the Larntz–Perlman procedure [36] for testing the equality of correlation matrices computed over non-overlapping subsamples: the pre-crisis and crisis periods in the group of markets investigated. Longin and Solnik [37] affirmed that the knowledge about international covariance and correlation matrices of asset returns and their behaviors is essential for the calculation of portfolios.

To examine the equality of correlation matrices, we can test the pair of hypotheses:

(6) $$H_{0}:P_{C}=P_{PC}\;H_{1}:P_{C}\neq P_{PC}$$

where $P_{C}$ and $P_{PC}$ are population correlation matrices in the turbulent and tranquil periods. Rejection of the $H_{0}$ indicates lack of equality of correlation matrices in a turbulent episode.

In this article, we used the test statistic proposed by Larntz and Perlman [36]:

(7) $$T_{LP}=\sqrt{\frac{n-3}{2}}*\underset{1\leq i<j\leq p}{\max}\left|z_{ij}^{C}-z_{ij}^{PC}\right|$$

where $z_{ij}^{C}$ and $z_{ij}^{PC}$ are the Fisher *z*-transformed correlation between ${\hat{\rho }}_{ij}^{C}$ and ${\hat{\rho }}_{ij}^{PC}$.

### 4.3. Mutual Information

Mutual information (MI) is a measure of statistical independence between two random variables, and it has its usage in evaluating both linear and nonlinear relationships [9]. Moreover, MI is defined as the amount of information transferred between studied systems [27].

There is no single commonly used MI estimator, but there are studies that compare them [38,39,40,41,42,43,44]. Determined by the sample size and underlying distribution or process, the MI rises with partition of an interval for time series. There are three main groups of estimators: histogram-based estimators, k-nearest neighbors, and kernel estimators [39,40]. Among histogram-based estimators we can distinguish three main subgroups: equidistant partitioning—bins of equal length [44]; equiprobable partitioning—each bin has the same occupancy, i.e., marginal equiquantization [45]; and adaptive partitioning as an extension of the previous two proposed by Darbellay and Vajda [41]. The k-nearest neighbors method takes into account the probability distributions for the distance between the point at which the density is to be estimated and its k-th nearest neighbor [40]. Another approach is to apply the kernel mutual information estimator constructed by Moon et al. [39] to centering kernel function at the data samples. According to the approach proposed by Darbellay [45], the marginal equiquantization estimation process allows one to maximize mutual information. Furthermore Dionísio et al. [46] emphasize that the comparison of MI is difficult in some contexts; therefore, it should apply a normalized measure of MI. Nevertheless, in order to ensure the comparability of our results with the study conducted by Gao and Mei [8], we will use the equidistant partitioning estimation process for our calculations.

In the study of MI, the selected method to discretize the time series is the binning method [9]. We fragmentize the range of the time series into n disjoint intervals $x_{n}\left(n=1,2,3,\ldots ,N;\;x_{n}=0,\;1,\;2,\;3\right)$ with fraction of all measurements equal to $p\left(x_{n}\right)=1/n$. By grouping the time series into bins $I:x_{n}\left(n=1,2,3,\ldots ,N;\;x_{n}=0,\;1,\;2,\;3\right)\;$and $J:y_{n}\left(n=1,2,3,\ldots ,N;\;y_{n}=0,\;1,\;2,\;3\right)$ that share identical length N, we create two discrete processes. The MI is given as:

(8) $$M\left(X;Y\right)=\sum_{x_{n},y_{n}}p\left(x_{n},y_{n}\right)log\frac{p\left(x_{n},y_{n}\right)}{p\left(x_{n}\right)p\left(y_{n}\right)}$$

### 4.4. Transfer Entropy

Transfer entropy (TE) was introduced by Schreiber [47] as an approach to measuring the direct exchange of the flow of information between two systems evolving in time. Considering two stationary and discrete processes $I:x_{n}\left(n=1,2,3,\ldots ,N;\;x_{n}=0,\;1,\;2,\;3\right)\;$and $J:y_{n}\left(n=1,2,3,\ldots ,N;\;y_{n}=0,\;1,\;2,\;3\right)$ that share identical length N, we measure the TE with J→I as the deviation of information collected from the previous state of I that comes purely from the latest state of I, which in turn was received from the last joint state of I and J [8,48]. The information propagation about the subsequent state of $x_{n+1}$ of I was received from the last joint state of I and J:

(9) $$h_{1}=-\underset{x_{n+1}}{\sum }p\left(x_{n+1},x_{n},y_{n}\right)*\log p(x_{n+1}|x_{n},y_{n})$$

The state of the subsequent observation $x_{n+1}$ of I is not based on the state of J; therefore, the information was received only from the state of I:

(10) $$h_{2}=-\underset{x_{n+1}}{\sum }p\left(x_{n+1},x_{n}\right)*\log p(x_{n+1}|x_{n})$$

The transfer entropy with processes J→I:

(11) $$T_{J\to I}=h_{2}-h_{1}=\underset{x_{n+1},x_{n},y_{n}}{\sum }p\left(x_{n+1},x_{n},y_{n}\right)*\log\frac{p(x_{n+1}|x_{n},y_{n})}{p(x_{n+1}|x_{n})}$$

### 4.5. Summary of Methods

We would like to use a variety of methods, such as the cross-correlation, volatility-adjusted cross-correlation, Larntz–Perlman procedure [36], and the mutual information and transfer entropy approaches, to separately analyze the correlation structures for testing the linear and nonlinear relationships in returns between selected markets. Each method has advantages and disadvantages.

There is a sizeable empirical literature that presents nonlinear effects in financial time series [9]. It is not possible to model such behavior in a sufficient manner using Pearson correlation, due to the fact that it explores only linear relationships, ignoring a meaningful amount of information [49]. For this reason, it would be favorable to model both linear and nonlinear information using different methods.

Mutual information has solid foundations in the mathematical concept of information theory and can be used to model both linear and nonlinear connections but is easily influenced by dependencies that are not found in the covariance [40]. On the other hand, MI does not provide directional or dynamical information because of its static, symmetric property [47]. Furthermore, the amount of received information relies on discretization algorithms and bin size [9]. In comparison to MI, transfer entropy is more adequate for detecting the direct exchange of information between two systems, but, as Kaiser and Schreiber [50] pointed out, no similar monotonic convergence seems to hold. In contrast to MI, transfer entropy is created to avoid static correlations due to the common input signals [47]. This tool is widely used due to its close relationship to the concept of Granger causality [51], which is the cause for combining two approaches (information-theoretic and predictive) to analyze directional relations between processes [52].

## 5. Results

### 5.1. Cross-Market Correlations

**Figure 1:**
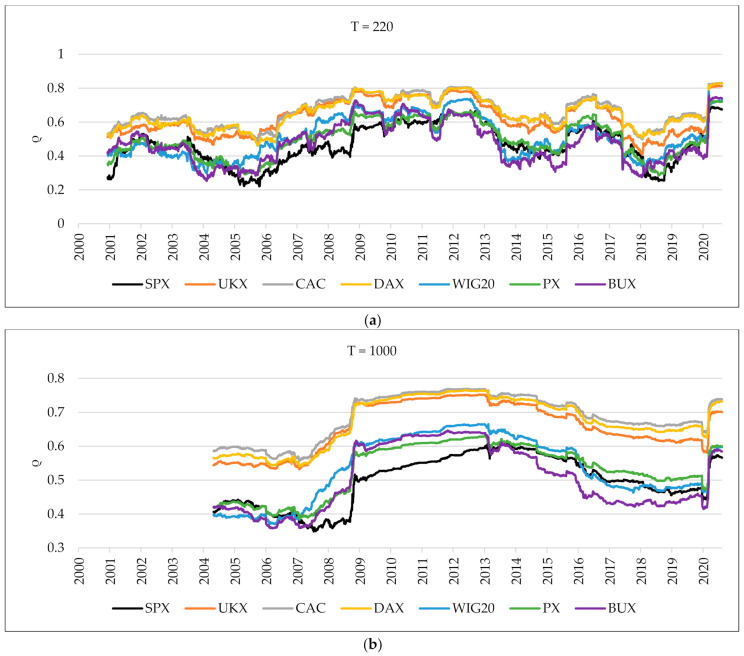
The mean linear correlations between each index and the rest of the indices using overlapping windows. The upper part is a 220-day fixed-size sliding window (**a**), and the one below is a 1000-day fixed-size sliding window (**b**).

In the first step, using linear correlations, we examine whether the degree of stock market connectedness between the US stock market and CEE differs from that in developed markets. Figure 1 shows the mean linear correlations between each index and the rest of the indices received by using overlapping windows. We split the time series into sequence based on the fixed-size sliding window of 220 days (up) and 1000 days (down), with 1 trading day window step length. After exploring different values, we identified the optimal parameters that ensure smoothly but dynamically changing results. Using various parameters of sliding windows allowed for receiving distinctive outputs that presented slightly different trends in the time series. The selected values are similar to Onnela, Chakraborti, Kaski, Kertész, and Kanto [10]. The mean linear correlations of the Western European markets are higher than in CEE indices. We can observe that UKX, CAC, and DAX indices move together throughout the complete sample, and the mean linear correlation of the CAC index is the highest. On the other hand, the mean linear correlation of the UKX index from 2016 (Brexit) to March 2020 (COVID-19 pandemic) has a weaker relationship with other Western European indices. The relationship between the mean linear correlations of CEE markets fluctuates during the whole period. In the time of the crisis, the mean linear correlation of the BUX index rose until 2013 and then dropped dramatically. Between 2009 and 2015, the mean correlation of the WIG20 is higher than other CEE indices. From 2016, the mean correlation of the PX index is higher than the WIG20 and BUX. Out of the CEE markets, the mean correlation of the BUX index increased the most during the COVID-19 pandemic. This evidence is consistent with the study on CEE indices during the COVID-19 period [17]. When the fixed-size sliding window is 220 days, the mean linear correlations of European markets bounce after falling in 2005, 2015, 2018, and in early 2020. The mean linear correlation of stock exchanges in the US (presented as a black line) declined from 2007 to 2009 and then began to rise again. Even with the 1000-day fixed size sliding window, it is still clear that the trend is going up, especially starting from March 2020.

**Figure 2:**
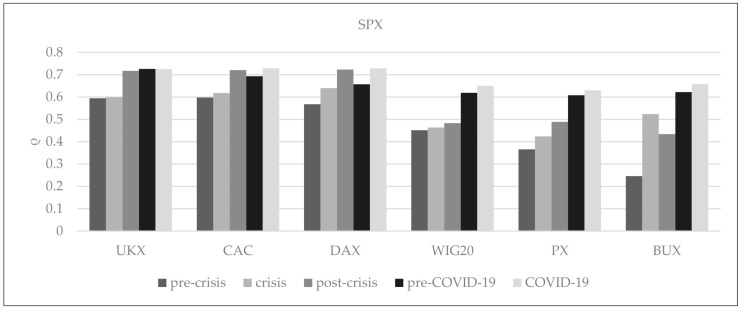
The linear correlations between the US and European stock market indices in the selected periods.

For further observation, the data were split into five short, distinctive periods: pre-crisis (1 September 2006 to 30 November 2007), crisis (1 December 2007 to 28 February 2009), post-crisis (1 March 2009 to 25 May 2010), pre-COVID-19 (30 September 2019 to 11 March 2020), and COVID-19 (12 March 2020 to 14 August 2020) in order to provide information on the strength and direction of the linear relationship. The results of the preliminary analysis are presented in Figure 2. We can see there that in all analyzed periods, linear correlations between the SPX and Western European indices achieve higher values than with CEE indices in all periods. The results show that COVID-19 has a considerable impact on all analyzed indices. The mean linear correlations of European and US markets prove to be higher during the COVID-19 period than in the crisis period. Furthermore, Western European indices are more affected by COVID-19 compared to CEE indices. During the COVID-19 period, the highest value of the correlation coefficient was observed in three cases: between the SPX and UKX, the SPX and CAC, and the SPX and DAX. In the group of CEE indices in the pre-crisis period, the linear correlation coefficients between the US and the WIG20 were at the highest level. During the crisis, this role is taken over by the BUX index; after the crisis, the PX index; and after that, during pre-COVID-19 and COVID-19 periods, again by the BUX index. Excluding the BUX index, all linear correlation coefficients between the US equity markets and selected European stock exchanges were higher in the post-crisis period than during and before the crisis. It is worth noting that only the linear correlation coefficient between the US equity markets and UKX index was lower in the COVID-19 period than in the pre-COVID-19 period.

Table 2 shows the standard contemporaneous cross-market correlations and adjusted correlation coefficients, as seen in (1), of daily logarithmic returns on pairs of indices—the SPX/stock market index. We take into consideration the dependencies in the complete sample (January 2000–mid-August 2020) as well as in two equally sized subsamples: the pre-crisis period, September 2006–November 2007 (290 days), and the crisis period, December 2007–February 2009 (290 days). We analyze the changes in cross-market linkages after the economic shock to the US financial market. The supporting values are equal to: ${\hat{\sigma }}_{C}^{2}=0.0006661542$ (the variance in the turbulent period in the US stock market) and ${\hat{\sigma }}_{PC}^{2}=0.0000864396\;$(the variance in the tranquil period in the US stock market), while the relative increase in the variance of the SPX returns, given by (2), is equal to δ=6.706584.

The results received in Table 2 for the crisis period indicate that the contemporaneous correlations between the US and other stock exchanges were higher than during the pre-crisis period, but the differences were low. In both periods, the values of contemporaneous correlations were higher in Western Europe than in CEE. The results of the Forbes and Rigobon methodology [33] show the absence of significant changes in cross-market linkages. The value of adjusted correlation between US and European stock markets decreased during crisis. There is no reason to reject the null hypothesis that states that there are no significant changes in the adjusted correlation for all analyzed markets. For this method as well, the values of adjusted correlations were higher in Western Europe than in CEE.

Moreover, we take into consideration the dependencies in the complete sample (January 2000–mid-August 2020) as well as in two equally sized subsamples: the pre-COVID-19 period of 30 September 2019–11 March 2020 (103 days) and the COVID-19 period of 12 March 2020–14 August 2020 (103 days). As shown in Table 3, we analyze the changes in cross-market linkages after the COVID-19 shock to the US financial market. The supporting values are equal to: ${\hat{\sigma }}_{C}^{2}=0.0008037915$ (the variance in the COVID-19 period in the US stock market) and ${\hat{\sigma }}_{PC}^{2}=0.0002521314\;$(the variance in the tranquil period in the US stock market), while the relative increase in the variance of the SPX returns, given by (3), is equal to δ=2.187987.

The results received in Table 3 for the COVID-19 period indicate that the contemporaneous correlations between the US and other stock exchanges (except UKX) were higher than during the pre-COVID-19 period; however, the differences were low. These results provide support for the theory of Ferreira, Dionísio, Almeida, Quintino, and Aslam [31] that the pandemic crisis may be a factor for the intensification of US indices. Similar results were obtained in the study by Czech, Wielechowski, Kotyza, Benešová, and Laputková [17] and Aslam et al. [53], who emphasize that the COVID-19 pandemic caused great impacts on CEE stock markets. In both periods, the values of contemporaneous correlations were higher in Western Europe than in CEE. For DAX, WIG20, PX, and BUX, we reject the null hypothesis, which suggests the existence of changes in correlation. On the other hand, the results of the Forbes and Rigobon methodology [33] show the absence of significant changes in cross-market linkages. The value of adjusted correlation between US and European stock markets decreased during the pandemic. There is no reason to reject the null hypothesis that states that there are no significant changes in the adjusted correlation for all analyzed markets. For this method as well, the values of adjusted correlations were higher in Western Europe than in CEE.

We observed that, compared to the 2007–2009 crisis, contemporaneous correlations between the US and other stock exchanges increased significantly during the pre-COVID-19 and COVID-19 periods (Table 2 and Table 3). In the case of the 2007–2009 crisis, we find one market (BUX) which indicates the lack of equality of correlation matrices, while during the COVID-19 period we find as many as four markets (DAX, WIG20, PX, BUX).

### 5.2. Larntz–Perlman Procedure

Table 4 summarizes the Larntz–Perlman test [36] performed on the SPX and the six European stock indices. We have reason to reject the null hypothesis (6), which suggests the stability of the correlation matrix via three adjacent sub-periods:

- the pre-crisis period, September 2006–November 2007 (290 days), and the crisis period, December 2007–February 2009 (290 days);
- the crisis period, December 2007–February 2009 (290 days), and the post-crisis period, March 2009–May 2010 (290 days); and
- the pre-COVID-19 period, 30 September 2019–11 March 2020 (103 days), and the COVID-19 period, 12 March 2020–14 August 2020 (103 days).

### 5.3. Mutual Information

**Figure 3:**
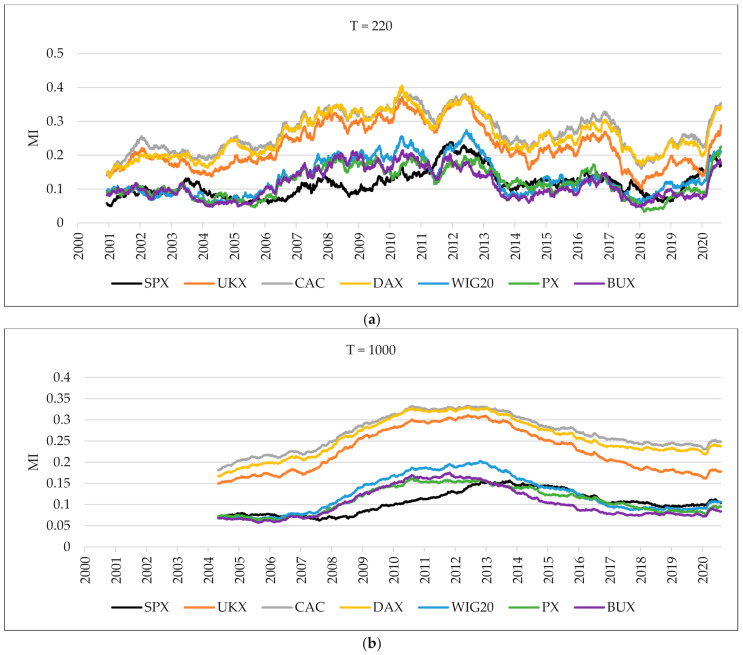
The average mutual information between each index and the rest of the indices using overlapping windows. The upper part is a 220-day fixed-size sliding window (**a**), and the one below is a 1000-day fixed-size sliding window (**b**).

**Figure 4:**
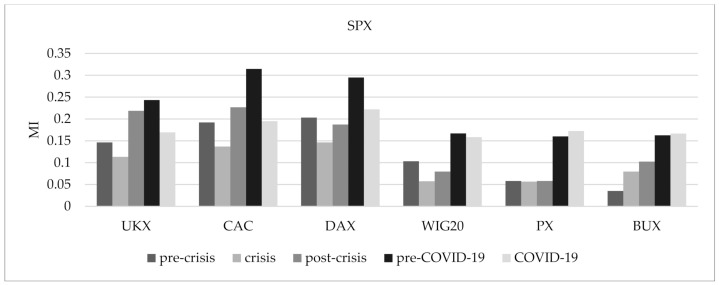
The mutual information between the US stock index and six European stock indices during the selected periods.

Figure 3 shows the outcome of average mutual information evolving in time. When the fixed-size sliding window equals 220 days, the average mutual information of European markets bounced after the fall that happened at the end of 2005, which is consistent with the mean linear correlation. Starting from March 2020, we can observe another soaring growth in the average mutual information of European markets. For the 1000-day fixed-size sliding window, the average mutual information showed an upward trend until 2013, when it peaked. It is worth noting that, starting from March 2020, we can see the growing tendency again; however, the UKX index is no longer so closely associated with other Western countries. Our main interest is in analyzing the connection between the US equity markets and European stock exchanges in the financial crisis of 2007–2009 and during the COVID-19 pandemic. The results received by comparing the MI in pre-crisis, crisis, and post-crisis periods are shown in Figure 4. Except for Hungary’s stock exchange, the MI between the US equity markets and other European stock indices is lower during the crisis in comparison to the pre-crisis period. We observe similar results for COVID-19 in comparison to the pre-COVID-19 period, except for Hungary’s and Czech Republic’s stock exchanges.

### 5.4. Transfer Entropy

**Figure 5:**
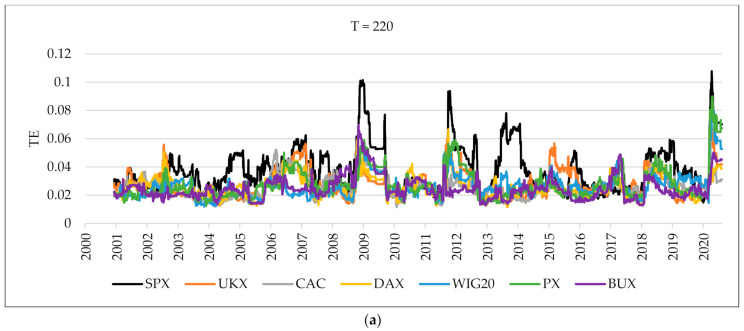

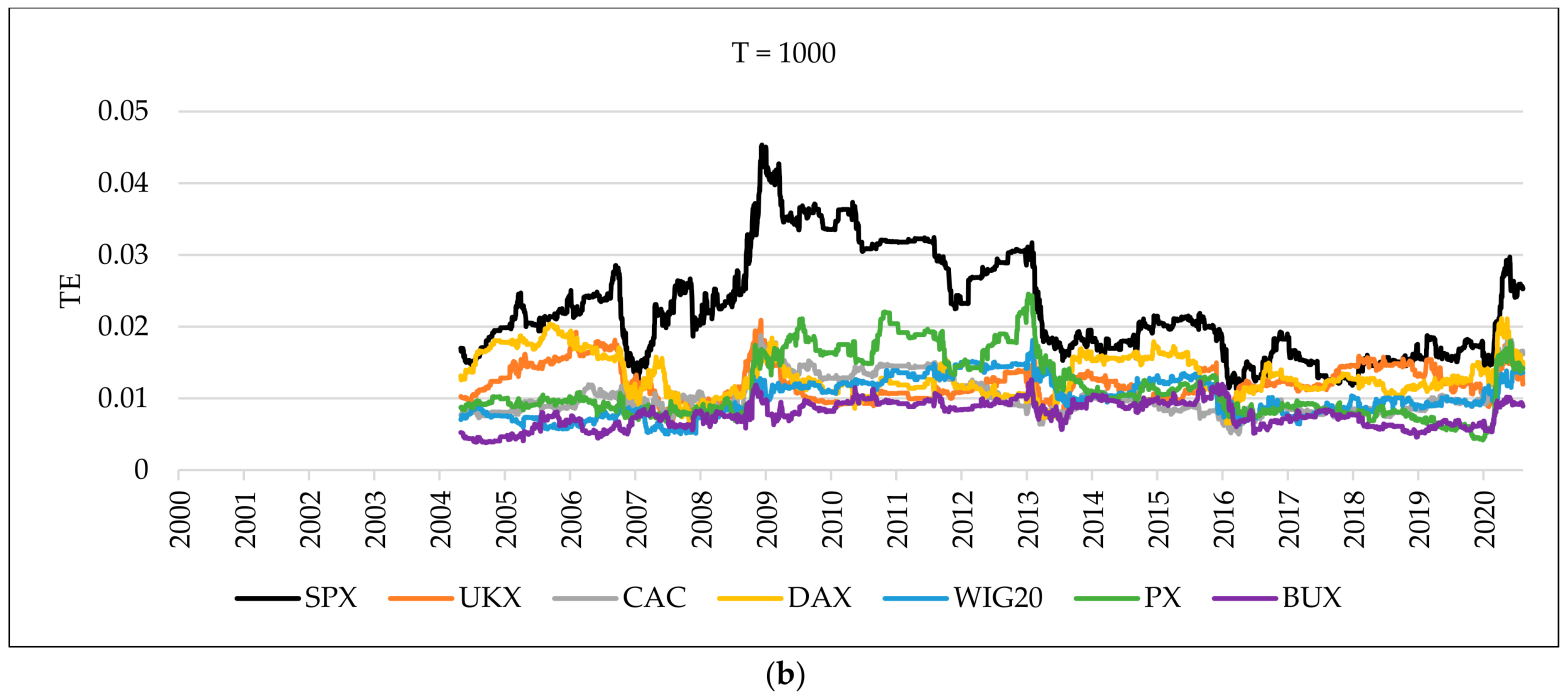
The average transfer entropy between each index and the rest of the indices using overlapping windows. The upper part is a 220-day fixed-size sliding window (**a**), and the one below is a 1000-day fixed-size sliding window (**b**).

**Figure 6:**
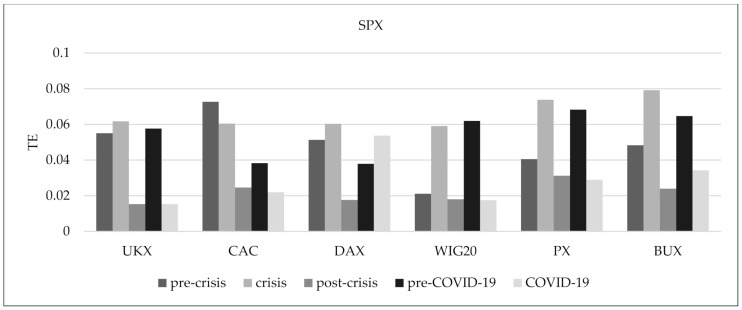
The transfer entropy from the US equity markets to six European equity markets during selected periods.

Figure 5 presents quickly changing outcomes of the average transfer entropy. We can observe that the average transfer entropy of the US stock market index reaches higher levels in comparison to the other markets. When the fixed-size sliding window is 220 days, the average transfer entropy of the US stock market index before January 2009 soars, but the peaks that it exhibits are sharp and narrow. A similar situation can be observed in March 2020. When the fixed-size sliding window is 1000 days, the average transfer entropy of the US stock market index grows continuously, then starts to decline after 2009, and rises again in March 2020. Figure 6 shows the outcomes of the TE values of the US equity markets of six European stock exchanges during the pre-crisis, crisis, post-crisis, pre-COVID-19, and COVID-19 periods. The TE from the US equity markets to Western Europe stock indices present higher values than CEE ones in the pre-crisis period. We observe the opposite situation in the pre-COVID-19 period. On the other hand, the TE from the US equity markets to CEE stock indices in the crisis period is higher than to Western Europe indices. In the COVID-19 period, the TE from the US equity markets to DAX and BUX stock indices was the highest. In the pre-crisis period, the TE from the US equity market to Poland is the weakest in comparison to other countries, but, during the crisis, it increased the most, reaching a level similar to Western Europe. On the other hand, in the pre-COVID-19 period, the TE from the US equity market to Germany is the weakest in comparison to other countries, but during the pandemic it increased the most. The TE from the US equity markets to selected European stock indices in the crisis period reaches a higher level in comparison to the pre-crisis period, with France being the exception. Contrary to that, the TE from the US equity markets to selected European stock indices in the COVID-19 period reaches lower levels in comparison to the pre-COVID-19 period, with Germany being the exception. During the crisis, the TE from the US equity markets to the BUX index is the highest in the group of CEE countries and the UKX index in the group of Western Europe. In the post-crisis period, the TE from the US equity markets to other indices decreased dramatically, especially the BUX and UKX indices. During COVID-19, the TE from the US equity markets to the BUX index is the highest in the group of CEE countries and the DAX index in the group of Western Europe. Based on the presented outcomes, we deduce that when the fixed-size sliding window equals 1000 days, the growth of mean linear correlations slows down considerably after 2009. At the same time, the average mutual information continues to rise until it peaks around 2013. Thus, we conclude that the stronger dependencies between all indices that can be observed after 2009 are due to the nonlinear effect. Similar results have been obtained by Gao and Mei [8] and Haluszczynski et al. [9].

### 5.5. Comparison of Results

We would like to model linear and nonlinear behavior in financial time series through the evaluation of information on dynamic correlations. Due to that, we used not only linear Pearson correlation but also mutual information, which can be used both for linear and nonlinear connections, as well as transfer entropy, which allows one to examine nonlinear connections.

Table 5 shows the comparison of different methods used to measure the dependence between the US stock index and selected European stock indices. For each of the three methods, we compute the values for pre-crisis, crisis, post-crisis, pre-COVID-19, and COVID-19 periods. The correlation coefficient values range from 0.246 to 0.729. In the case of examined countries, there is a clear separation between two strongly connected groups: Western European indices and CEE indices. We recognize that Western Europe has higher linear correlation coefficient values (from 0.568 to 0.729) than CEE (from 0.246 to 0.658). The levels of correlation increased significantly in the pre-COVID-19 and COVID-19 periods in all markets (the highest for CAX and DAX indices from Western Europe and for PX and BUX from CEE in the COVID-19 period). The results confirm that the COVID-19 pandemic has led to a growth in European financial market risk, which is in line with Zhang et al. [12], Akhtaruzzaman et al. [13], Shehzad et al. [54], and Zaremba et al. [14]. It should be stressed that the amplitude of growth was much higher in CEE markets, which is similar to the findings of Topcu and Gulal [16] and Tilfani, Ferreira, and Boukfaoui [55]. The most stable level of correlation in all analyzed periods is presented by the UKX index (from 0.595 to 0.726) and the CAC index (from 0.598 to 0.729). On the other hand, BUX increased the most between pre-crisis and crisis periods (from 0.246 to 0.524). After the crisis, BUX began to behave like other CEE countries. Overall, relationships between local centers are greater within these groups than between them. These results are in line with those obtained by Stoica and Diaconașu [56] and Gradojević and Dobardžić [57]. The results demonstrated that regional market integration is strengthened in times of crisis or pandemic.

As can be observed in Table 5, similar conclusions to those received by using linear correlation can be obtained with mutual information. For both methods, Western Europe is the region that attains the largest values. Furthermore, the highest values of mutual information are achieved in the pre-COVID-19 period for Western Europe and in the COVID-19 period for CEE regions. It is interesting to note that the transfer entropy presents slightly different results. The values of transfer entropy in CEE are higher (from 0.017 to 0.079) than in Western Europe (from 0.015 to 0.025), which can be especially observed in the crisis and pre-COVID-19 periods. As per our results, notable information cannot be expressed well by linear measure, hence the usage of different methods that intercept linear and nonlinear correlations. In conclusion, our analysis suggests that stock indices quickly responded to the GFC as well as the COVID-19 pandemic, and these responses changed over time depending on the information flowing through markets.

## 6. Discussion and Conclusions

This study provides an analysis of the effect of the GFC and the COVID-19 pandemic on European stock markets. The main goal of this paper is to compare the risk transfer between US stock market indices and six European stock market indices before, during, and after the GFC, as well as before and during the COVID-19 outbreak. In our study, we also emphasize the differences in the correlation structure between CEE and Western European markets. We used a variety of methods to separately analyze the correlation structures for testing the linear and nonlinear structure of relationships in returns between the US stock index and selected European stock indices.

Testing the connectedness during the crisis period, the correlation between SPX and CEE indices changed more in terms of growth than in Western European indices. This is only a partial confirmation of earlier research [7], stating that the CEE stock exchanges are not more vulnerable to contagion, even if they are less liquid than Western European markets. Additionally, our findings stress that the amplitude of growth in the pre-COVID-19 period is much higher in CEE markets. Given a slower economic growth and relatively not liquid capital markets, emerging markets have probably limited resources to cope with the pandemic.

Nevertheless, the relationship between the mean linear correlations of CEE markets fluctuates during the whole period. In the years 2009–2015, the mean linear correlation for WIG20 is higher than for other CEE indices, but, starting from 2016, the correlation index for the PX is higher than for the WIG20 and the BUX. In the analyzed period, the stock markets in CEE were not stable or resistant to crisis shocks. This result may be explained by the smaller integration of CEE stock markets with global capital markets. For investors, this means another source of risk diversification in CEE markets.

Comparing to the GFC, our findings emphasize that the linear correlations between the S&P 500 and all European indices increased significantly in the pre-COVID-19 period. The negative impact of COVID-19 on stock markets continued or slightly increased by mid-August 2020. The results show that the COVID-19 pandemic has led to a growth in European financial market risk. These findings confirm those of earlier studies, such as Ferreira [58] and Grabowski [59]. An analysis of the volatility spillovers indicates that CEE markets are the recipients of volatility. As opposed to the previous research of Topcu and Gulal [16], our findings do not confirm that the influence of COVID-19 on emerging stock markets has gradually fallen and began to taper off by mid-April 2020.

The results that we obtained indicate that there are relatively significant differences between linear and nonlinear estimation. The transfer entropy from the US equity markets to CEE stock indices during the crisis is higher than to Western Europe indices. Before the crisis, the transfer entropy from the US equity market to Poland is the weakest compared to other countries, but, during the crisis, it increased the most. During the crisis, the transfer entropy from the US equity market to Poland is similar to Western Europe. Additionally, we infer that nonlinear effects lead to stronger dependencies between all indices after 2009. Starting from the COVID-19 pandemic period, we can observe soaring growth in the average mutual information and transfer entropy of all European markets.

Our study of European stock markets shows that cases of intensified and broken links between markets are particularly visible in CEE countries. This evidence may suggest that emerging equity markets are increasingly integrated into mature markets, thus becoming dependent on certain crises and pandemic outbreaks. This may be explained by short-term capital flows from less stable markets, changing political circumstances. Undoubtedly, research should provide an interesting insight for potential investors diversifying their stock portfolio. Our research has implications for risk management and asset pricing. Although CEE countries are considered a homogeneous group by international investors, the financial markets of these countries show varying degrees of integration. Therefore, from a portfolio diversification perspective, less developed markets may offer risk diversification opportunities that investors can capitalize on. For the purpose of portfolio risk management, information about the linkages between markets can be important for investors in making decisions. In addition, information on the increasing connectedness between markets may be relevant when portfolios are reallocated.

We believe that this study may be a benchmark for financial market network structure for further research in this area. Therefore, future researchers should test whether the results remain insignificant over a longer time horizon. Additionally, similar to the vast majority of research on contagion in emerging economies, our research focuses on the analysis of daily and weekly data. However, it would be worthwhile to investigate the connectedness of European stock markets with high-frequency information.

## Figures and Tables

**Table 1:** Summarized statistics for daily returns.

Index	Mean	Standard Deviation	Skewness	Excess Kurtosis	Doornik–Hansen Test
SPX	0.0001839	0.0131	−0.482 [0.000]	10.584 [0.000]	4805.214 [0.000]
UKX	−0.0000148	0.0124	−0.289 [0.000]	7.956 [0.000]	1515.870 [0.000]
CAC	−0.0000208	0.0152	−0.297 [0.000]	6.630 [0.000]	1892.501 [0.000]
DAX	0.0001436	0.0156	−0.251 [0.000]	5.931 [0.000]	2270.681 [0.000]
WIG20	0.0000092	0.0157	−0.288 [0.000]	5.111 [0.000]	819.241 [0.000]
PX	0.0001328	0.0143	−1.041 [0.000]	19.041 [0.000]	7536.307 [0.000]
BUX	0.0003067	0.0156	0.123 [0.000]	13.298 [0.000]	3660.866 [0.000]

**Table 2:** Contemporaneous cross-correlations and adjusted correlations of daily logarithmic returns in pairs—the SPX/stock market index—subsamples: the pre-crisis and crisis.

Index	Contemporaneous Cross-Correlations						Adjusted Correlations ([33])			
	Complete Sample (1)	Pre-Crisis (2)	Crisis (3)				Crisis (3)			
	$\hat{\rho }$	${\hat{\rho }}_{PC}$	${\hat{\rho }}_{C}$	Change Compared to the Period (2)	*Z*-Statistic	Hypothesis	${\hat{\rho }}_{VA}$	Change Compared to the Period (2)	*Z*-Statistic	Hypothesis
UKX	0.598 [0.000]	0.595 [0.000]	0.600 [0.000]	0.008	0.089	H0	0.261	−0.562	−5.011	H0
CAC	0.615 [0.000]	0.598 [0.000]	0.618 [0.000]	0.034	0.382	H0	0.273	−0.544	−4.917	H0
DAX	0.634 [0.000]	0.568 [0.000]	0.640 [0.000]	0.127	1.366	H0	0.287	−0.494	−4.175	H0
WIG20	0.407 [0.000]	0.452 [0.000]	0.464 [0.000]	0.027	0.182	H0	0.185	−0.590	−3.586	H0
PX	0.382 [0.000]	0.366 [0.000]	0.424 [0.000]	0.157	0.816	H0	0.166	−0.546	−2.592	H0
BUX	0.395 [0.000]	0.246 [0.000]	0.524 [0.000]	1.130	3.960	H1	0.216	−0.121	−0.376	H0

Notes: The table presents the data received through the analysis of the complete sample period of January 2000–December 2019 (4623 days); the pre-crisis period of September 2006–November 2007 (290 days); and the crisis period of December 2007–February 2009 (290 days). The numbers in brackets are *p*-values. Fisher *Z*-statistic tests were null for no changes in correlation. Critical value of Student’s t distribution is 1.711 (at the 10% significance level).

**Table 3:** Contemporaneous cross-correlations and adjusted correlations of daily logarithmic returns in pairs—the SPX/stock market index—subsamples: pre-COVID-19 and COVID-19.

Index	Contemporaneous Cross-Correlations						Adjusted Correlations ([33])			
	Complete Sample (1)	Pre-COVID-19 (2)	COVID-19 (3)				COVID-19 (3)			
	$\hat{\rho }$	${\hat{\rho }}_{PC}$	${\hat{\rho }}_{C}$	Change Compared to the Period (2)	Z-Statistic	Hypothesis	${\hat{\rho }}_{VA}$	Change Compared to the Period (2)	Z-Statistic	Hypothesis
UKX	0.598 [0.000]	0.726 [0.000]	0.725 [0.000]	−0.001	1.651	H_0_	0.508	−0.300	−0.882	H_0_
CAC	0.615 [0.000]	0.693 [0.000]	0.729 [0.000]	0.051	1.674	H_0_	0.512	−0.261	−0.878	H_0_
DAX	0.634 [0.000]	0.657 [0.000]	0.729 [0.000]	0.110	1.990	H_1_	0.512	−0.221	−0.559	H_0_
WIG20	0.407 [0.000]	0.619 [0.000]	0.650 [0.000]	0.050	2.046	H_1_	0.432	−0.302	−0.169	H_0_
PX	0.382 [0.000]	0.608 [0.000]	0.630 [0.000]	0.036	2.529	H_1_	0.414	−0.319	0.397	H_0_
BUX	0.395 [0.000]	0.622 [0.000]	0.658 [0.000]	0.058	3.805	H_1_	0.440	−0.293	1.560	H_0_

Notes: The table presents the data received through the analysis of the complete sample period of January 2000–mid-August 2020 (4773 days); the pre-COVID-19 period of 30 September 2019–11 March 2020 (103 days); and the COVID-19 period of 12 March 2020–14 August 2020 (103 days). The numbers in brackets are *p*-values. Fisher *Z*-statistic tests were null for no changes in correlation. Critical value of Student’s t distribution is 1.711 (at the 10% significance level).

**Table 4:** Results of the Larntz–Perlman test.

Test Periods	Larntz–Perlman Test				
	$\mathrm{Test}\;\mathrm{Statistic}\;T_{LP}$	$b_{\alpha }\;\mathrm{Critical}\;\mathrm{Value}\;(5\%)$		$b_{\alpha }\;\mathrm{Critical}\;\mathrm{Value}\;(10\%)$	
September 2006–November 2007 and December 2007–February 2009	5.257	2.63	$H_{0}$	2.38	$H_{0}$
December 2007–February 2009 and March 2009–May 2010	3.076	2.63	$H_{0}$	2.38	$H_{0}$
30 September 2019–11 March 2020 and 12 March 2020–14 August 2020	3.006	2.63	$H_{0}$	2.38	$H_{0}$

**Table 5:** Comparison of different methods used to measure the dependence between the US stock index and European indices during pre-crisis, crisis, post-crisis, pre-COVID-19, and COVID-19 periods.

Period	Group of Countries	Index	Linear Correlations	Mutual Information	Transfer Entropy
Pre-crisis	West Europe	UKX	0.595	0.146	0.055
		CAC	0.598	0.192	0.073
		DAX	0.568	0.203	0.051
	CEE	WIG20	0.452	0.103	0.021
		PX	0.366	0.058	0.041
		BUX	0.246	0.035	0.048
Crisis	West Europe	UKX	0.600	0.113	0.062
		CAC	0.618	0.137	0.060
		DAX	0.64	0.146	0.060
	CEE	WIG20	0.464	0.058	0.059
		PX	0.424	0.057	0.074
		BUX	0.524	0.080	0.079
Post-crisis	West Europe	UKX	0.717	0.219	0.015
		CAC	0.721	0.227	0.025
		DAX	0.723	0.187	0.018
	CEE	WIG20	0.483	0.080	0.018
		PX	0.489	0.058	0.031
		BUX	0.434	0.103	0.024
Pre-COVID-19	West Europe	UKX	0.726	0.243	0.058
		CAC	0.693	0.315	0.038
		DAX	0.657	0.295	0.038
	CEE	WIG20	0.619	0.167	0.062
		PX	0.608	0.160	0.068
		BUX	0.622	0.163	0.065
COVID-19	West Europe	UKX	0.725	0.169	0.015
		CAC	0.729	0.195	0.022
		DAX	0.729	0.222	0.054
	CEE	WIG20	0.650	0.159	0.017
		PX	0.630	0.173	0.029
		BUX	0.658	0.167	0.034

Notes: The rows of a heat map represent stock indices in specific periods, and the columns represent the methods used to measure the dependence between the US stock index and six European stock indices during pre-crisis, crisis, post-crisis, pre-COVID-19, and COVID-19 periods. Each cell in the particular methods is colorized based on the values (from green for the lowest values to red for the highest ones).

## Data Availability

All the data supporting reported results come from: https://stooq.pl (accessed on 9 February 2022).
